# The odds of duplicate gene persistence after polyploidization

**DOI:** 10.1186/1471-2164-12-599

**Published:** 2011-12-12

**Authors:** Frédéric JJ Chain, Jonathan Dushoff, Ben J Evans

**Affiliations:** 1 Department of Biology, McMaster University, 1280 Main Street West, Hamilton, ON, L8S 4K1, Canada; 2 Department of Evolutionary Ecology, Max Planck Institute for Evolutionary Biology, August-Thienemann-Str. 2, 24306 Plön, Germany

## Abstract

**Background:**

Gene duplication is an important biological phenomenon associated with genomic redundancy, degeneration, specialization, innovation, and speciation. After duplication, both copies continue functioning when natural selection favors duplicated protein function or expression, or when mutations make them functionally distinct before one copy is silenced.

**Results:**

Here we quantify the degree to which genetic parameters related to gene expression, molecular evolution, and gene structure in a diploid frog - *Silurana tropicalis *- influence the odds of functional persistence of orthologous duplicate genes in a closely related tetraploid species - *Xenopus laevis*. Using public databases and 454 pyrosequencing, we obtained genetic and expression data from *S. tropicalis *orthologs of 3,387 *X. laevis *paralogs and 4,746 *X. laevis *singletons - the most comprehensive dataset for African clawed frogs yet analyzed. Using logistic regression, we demonstrate that the most important predictors of the odds of duplicate gene persistence in the tetraploid species are the total gene expression level and evenness of expression across tissues and development in the diploid species. Slow protein evolution and information density (fewer exons, shorter introns) in the diploid are also positively correlated with duplicate gene persistence in the tetraploid.

**Conclusions:**

Our findings suggest that a combination of factors contribute to duplicate gene persistence following whole genome duplication, but that the total expression level and evenness of expression across tissues and through development before duplication are most important. We speculate that these parameters are useful predictors of duplicate gene longevity after whole genome duplication in other taxa.

## Background

Gene duplication is a fundamental genomic process that occurs on a small scale via segmental duplication and on a large scale via whole genome duplication (WGD). Gene duplication triggers biological innovation (neofunctionalization; 1), reduces pleiotropy by division of labor (subfunctionalization; 2), contributes to reproductive incompatibilities via divergent resolution [3], and generates redundancy [4]. More often than not, however, the ultimate fate of duplicate genes is the loss of one copy, usually within a few million years [5]. Persistence of duplicate genes occurs when natural selection favors duplicated protein function or expression, or when modifications soon after duplication distinguish the duplicates in some way (for example by causing paralogous expression domains to diverge). The targets of natural selection after gene duplication can be broadly divided into those that involve no change in function, gain of function, or loss of function [6]. For example, both copies of a duplicated gene may evade pseudogenization without changed function if having higher expression is advantageous, if disrupting the dosages of interacting duplicated proteins is disadvantageous, or if segregation of functionally distinct alleles that evolved prior to duplication is beneficial [5,7-9]. Persistence of duplicates could also be triggered by changes after duplication if advantageous mutations occur in one or both paralogs, or if degenerative mutations make the paralogs non-redundant [1,2,10,11]. Over time, increased divergence between duplicate genes reduces the chances of synfunctionalization, a phenomenon by which one of the paralogs convergently evolves the function or expression domain of the other, thereby re-establishing redundancy and leading to the loss of one paralog [12]. A challenge in evolutionary biology, then, is to identify the relative importance of different mechanisms for duplicate gene retention, and also to understand what genetic factors prior to duplication portend a high probability of both paralogs persisting as functional loci after duplication.

Various factors are correlated with the odds of duplicate gene persistence, including how paralogs are generated (by segmental duplication or WGD), the breadth and intensity of expression, dosage sensitivity, interactions with other proteins, the number of functional domains, and the rate of evolution [13-23]. In the case of *ohnologs *- duplicate genes generated by WGD, expression dosage is thought to frequently drive functional persistence, suggesting that the stoichiometry of expression among duplicated loci is important [21,24-29]. However, many of these factors are correlated with each other, making it challenging to tease apart their relative impact. For example, genes with high expression intensities or broad expression patterns tend to evolve slowly [30-37] so it is not clear with which degree each variable influences duplicate gene persistence after factoring out the role of the other. Our focus here is to disentangle and quantify the relative contributions of several variables to duplicate gene persistence after WGD.

### *Xenopus *as a model to study duplicate genes

Whole genome duplication occurred in an ancestor of African clawed frogs of the genus *Xenopus *~ 21 - 41 million years ago, after divergence from ancestors of the genus *Silurana *[6,38]. This essentially duplicated all loci in the nuclear genome, generating ohnologs. Here we build on previous studies of the molecular evolution and expression of duplicate genes in *X. laevis *(reviewed in 39) by assembling and analyzing the most comprehensive dataset yet from this species in terms of the number of genes and the scope of genetic parameters. Using data from the diploid species *S. tropicalis*, we use logistic regression to evaluate the odds that the orthologous paralogs in the tetraploid species *X. laevis *both remain functional. Logistic regression allows us to jointly quantify the relative impact of multiple genetic variables from a diploid genome on the odds of duplicate persistence in a tetraploid genome, while factoring out statistical noise caused by post-WGD changes in the tetraploid [40].

## Methods

This study examines gene expression, molecular evolution, and gene structure information of singleton genes in the diploid species *S. tropicalis *that are orthologous either to a pair of ohnologs in the tetraploid species *X. laevis *or to a single-copy gene in *X. laevis*. We assume that all single-copy genes in *X. laevis *were once part of a pair of ohnologs but that one copy has been lost due to post-WGD pseudogenization. Our analyses therefore consist of two gene sets: (1) gene triads, which include a pair of *X. laevis *ohnologs and the corresponding *S. tropicalis *singleton ortholog, and (2) gene dyads, which include one *X. laevis *singleton and the corresponding *S. tropicalis *singleton ortholog.

Nucleotide sequences from 3,387 gene triads and 4,746 dyads were gathered from the NCBI UniGene databases using tBLASTx and a reciprocal best hit approach. UniGenes are a set of non-redundant clusters of transcript sequences that compose the expressed sequence tag (EST) libraries. In a gene triad, the two *X. laevis *UniGenes were reciprocal best hits within the *X. laevis *UniGenes, and they both returned the same *S. tropicalis *UniGene as the top hit. In a dyad, each putative *X. laevis *singleton has a single unique reciprocal top hit with *S. tropicalis*. UniGenes that had more than two *X. laevis *genes or more than one *S. tropicalis *gene with reciprocal best hits were excluded. To test whether our putative singletons were indeed singletons, we tried to amplify the other ohnolog of 17 *X. laevis *singletons using PCR primers designed to amplify both the *X. laevis *singleton and the *S. tropicalis *ortholog [41]; in all cases only one gene copy was amplified in *X. laevis*. Triads and dyads were aligned using MUSCLE [42], and Perl scripts were used to predict the beginning and end of coding regions by looking for the longest open reading frame in either direction. Overlapping alignments shorter than 201 nucleotides were discarded along with 5' and 3' untranslated regions and indels.

Non-normalized EST libraries were obtained from NCBI (dbEST Library IDs: 10829, 10830, 10895, 10896, 20954, 20886, 21298, 20560, 20561, 20562, 20892, 20891, 20911, 20931, 20912, 20947, 20953, 20901, 16856, 16857, 16858, 16871, 16854, 16853, 16863, 16862, 16870, 16864, 16872, 17807, 16868, 16869, 16867, 16865, 16866, 17804, 17805, 17806, 16855, 16873, 16876, 16875, 16877, 16878, 16874, 16801, 16859, 16860, 16861, 16880, 8773, 8701, 20682, 9909, 9665, 9908, 14603). We classified and combined 718,484 *S. tropicalis *ESTs (from a total of 1,271,375) into 14 distinct adult tissues and 4 embryological stages: brain, bone, eye, heart, kidney, liver, lung, thymus, pancreas, skin, spleen, fat body, ovary, testis, egg, gastrula stage, neurula stage, and embryo stage 62. Each library consists of at least 3,900 transcripts (on average 39,915) and each individual UniGene has its own set of unique ESTs. We used the proportion of transcripts of each gene divided by the number of transcripts in a given EST library as an estimate of its expression level to control for the different sizes of the EST libraries. For twenty-three outlier genes with high expression, we truncated the expression level to a value of 1% of the respective EST library to prevent these outliers from dominating the results. Of the set of genes for which sequence data were available, a total of 3,298 triads and 4,426 dyads also had expression data (at least one *S. tropicalis *EST read in at least one EST database).

### Expression summary statistics

To characterize expression patterns in the diploid species *S. tropicalis*, three non-independent summary statistics were calculated: total expression (*T*), expression intensity (*I*), and expression evenness (*E*). Total expression *T *is simply the expression level (the proportion of total EST reads of a gene in a given EST library, *L*_i_) summed across all EST libraries (*T *= Σ *L*_i_). Genes that are evenly expressed at moderate levels in many tissues have similar *T *to genes that are highly expressed in only a few tissues. We therefore introduced a measure of "intensity". Intensity is the mean expression level as seen by a gene, rather than by a tissue. Thus, we expect a gene that is highly expressed in only a few tissues to have a moderate total expression, but a high level of intensity. We calculate intensity as a weighted average of expression levels, where the expression levels themselves are the weights: *I *= Σ *L*_i_^2 ^/Σ *L*_i_.

Although *T *and *I *capture the desired information about gene expression levels and distribution, we added a measure of evenness to give our linear model more flexibility. We define the evenness *E *as *T*/*I*; this is a logical measure of the "effective" number of tissues in which a gene is expressed, and we consider this analogous to how broadly a gene is expressed. *E *is equal to (Σ *E*_i_)^2 ^/Σ *E*_i_^2^, and is therefore Simpson's diversity (equivalent to 1/Simpson's index). *E *therefore measures how evenly distributed gene expression is across different tissues. A gene with relatively even distribution across tissues will have an elevated *E *(will be broadly expressed), irrespective of the total expression level. *T *was calculated for 8,133 genes. For 409 genes that had no EST reads, *T *was zero, *E *was undefined and set as missing, and *I *was undefined but set to zero since it must approach zero as expression levels approach zero.

### Molecular evolution

We used 454 pyrosequencing of cDNA from *Pipa carvalhoi *and *Hymenochirus curtipes *to generate outgroup sequences for analysis of molecular evolution in *Silurana *and *Xenopus *(following the same protocol as in [43]). Sequences were assembled using gsAssembler and gsMapper (454/Roche) and the resulting contigs aligned to the triads and dyads using Perl scripts, BLAST and MUSCLE. This effort recovered sequences from portions of 2,157 genes from *P. carvalhoi *or *H. curtipes *that were used to root a phylogeny with sequences from *S. tropicalis *and either one ortholog or both ohnologs of *X. laevis*. New sequences from the outgroup species have been deposited in the Transcriptome Shotgun Assembly database (JP285961 - JP288098, JP297711 - JP297788).

We calculated rates of nonsynonymous (dN) and synonymous (dS) substitutions per site, and the dN/dS rate ratio of the *S. tropicalis *lineage. These statistics were calculated with the codeml program in the PAML package version 3.15 [44] using a maximum likelihood model that individually estimates each type of substitution rate for each branch. Sixty-five values of dN/dS were undefined due to a dS of zero. To cope with this, we instead used an adjusted dN/dS in our analysis, defined as dN/(dS+0.02). We made this adjustment a priori (looking only at dS values) and performed it on all genes for which we had sequence data. By choosing adjusted dN/dS as our measure of the relative strength of selection in our model, we are simply choosing a different proxy for this underlying effect so that we are able to make use of more of our data.

### Gene structure

Using JGI annotations for *S. tropicalis *EST data, we collected statistics on gene structure for 6,075 genes. These statistics relate to content and packaging of information; for each gene we scored the number of exons, the length of the protein-coding region, the total length of introns and the amino acid diversity (Shannon Index).

### Logistic regression, missing data and confidence intervals

Logistic regression is a generalized linear model used for binomial regression, providing a useful statistical framework with which to explore the impact of continuous and potentially non-independent variables on a binary outcome (here, whether or not both copies of a duplicate gene persist after WGD). Logistic regression was recently employed to identify predictors of paralog retention originating from both WGD and tandem duplications in *Populus *[45]. Whereas that study used genetic variables taken from the tetraploid species, we used the logistic regression to test the association between genetic variables measured in a diploid and the persistence of orthologous ohnologs in a tetraploid. Our goal was not to make a predictive model, but to use the linear model as a tool to study associations and make inferences about evolutionary mechanisms. This analysis was performed using R [46].

We divided each of our variables by its standard deviation; thus the regression coefficients are proportional to the overall estimated importance of an approximation of the relative influence that each variable has on the outcome - the odds of persisting as a duplicate gene. Some of our variables have missing data. To make possible a single analysis that jointly considered all of the data, we substituted each missing value with the grand mean of that variable. Variables were not re-normalized after substitution because this would affect the variance and inferred importance of the variable in the logistic regression. This substitution allows us to jointly consider all available data in one analysis. Because this replacement affects the standard regression assumption, we report P-values based on a permutation test of the model. For each variable, we fitted the model 2000 times - once with the original data, and 1999 times with the focal variable replaced by a random permutation of itself. The two-tailed permutation P-value is twice the proportion of these fits (including the original) whose coefficients for the focal variable are greater (respectively, less than) or equal to a positive (negative) coefficient from the original fit. Since we conservatively count the original fit, our two-tailed P-value from 2000 tests cannot be less than 0.001. The permutation P-values are similar to the standard P-values from the logistic regression (not shown) and in fact give exactly the same pattern of significance. An analysis including the 2,021 genes without missing data was also performed and does not change our overall conclusions: one variable is no longer significant (number of exons) and another is now significant (amino acid diversity). The direction of the correlation of our significant variables does not change, and the relative strength of the strongest predictors (total expression, evenness of expression, dN and dS) remains the same.

## Results

To what degree do different genetic variables in a diploid species influence the probability of duplicate gene persistence in a closely related species after WGD? Table 1 presents the results of logistic regression between 10 variables in the diploid species *S. tropicalis *on the outcome of duplicate gene persistence in the tetraploid species *X. laevis*. A positive correlation indicates a positive interaction between the predictor variable in the diploid species and persistence in the tetraploid species. Our results indicate that several characteristics of gene expression, molecular evolution, and gene information content in *S. tropicalis *are significantly associated with whether or not genes duplicated by WGD persist in *X. laevis*.

**Table 1:** The logistic regression coefficients of each variable, their standard errors and the associated P-values from permutation tests.

Variable	Coefficient	Standard Error	P-value	
***T***	1.038	0.184	0.001	*

***I***	-0.017	0.086	0.477	

***E***	0.470	0.036	0.001	*

***dN***	-0.310	0.070	0.001	*

***dS***	-0.191	0.057	0.001	*

***dN/dS_adj***	-0.091	0.077	0.074	

**No.Exons**	-0.097	0.049	0.002	*

**Protein Length**	-0.052	0.048	0.074	

**Intron Length**	-0.151	0.037	0.001	*

**AA Diversity**	-0.022	0.024	0.351	

Variables include expression summary statistics Total *(T)*, Intensity *(I)*, and Evenness (*E*), the rate of nonsynonymous (*dN*) and synonymous (*dS*) subsitutions per site, the ratio of *dN*/*dS *after adjusting *dS *(*dNdS_adj*), the number of exons (No.Exons), the total protein-coding length in nucleotides (Protein Length), the total intron length (Intron Length) and the amino acid diversity (AA Diversity). See Methods for description of these variables. The coefficient column represents the change in the log odds of duplicate gene retention per unit increase in the predictor variable. A positive (respectively, negative) coefficient indicates a positive (negative) correlation between that variable in the diploid species *S. tropicalis *and the probability that a duplicate is retained in the tetraploid species *X. laevis*. Significant values of P < 0.01 are indicated with an asterisk.

### Gene Expression

Total expression (*T*) is the strongest predictor of duplicate gene functional persistence after WGD out of the parameters we considered. Expression evenness (*E*) is the second strongest predictor. These correlations are of significantly larger magnitude than all or most of the other parameters. This indicates that genes with high overall expression and even distribution across tissues and developmental stages tend to persist as duplicates following WGD. A third expression summary statistic (*I*) does not have a significant effect on the odds of persistence in our full model; since our three expression statistics are not independent, this does not mean intensity is not important, only that whatever effect intensity has is captured better by using T than I as a predictor in a linear model.

### Molecular Evolution

Two out of three non-independent variables related to molecular evolution are also significantly associated with persistence. The rate of nonsynonymous (dN) and synonymous (dS) substitutions are significantly negatively correlated, indicating that genes evolving slowly before WGD at both the protein level and overall, are more likely to persist after WGD. Selective constraint (dN/dS), however, does not have a significant effect on duplicate gene persistence when combined with our other variables.

### Gene Structure

Two out of four variables related to the information content and packaging of genes are also significantly associated with paralog persistence. Genes with fewer exons (thus fewer introns) and shorter total intron length are more likely to be retained after WGD in *X. laevis*. Under the scenario that the number of exons and intron length reflect how "efficiently" information is packaged in a gene, there is a positive association between efficient information packaging in *S. tropicalis *(fewer exons and shorter introns) and ohnolog retention in *X. laevis*. This also suggests that genes with a smaller mutational target are more likely to persist. The coefficients of the number of exons and the total intron length are both significantly lower in magnitude than *T *and *E *and also lower than dN and dS. The total protein length and the amino acid diversity, however, did not have significant correlations in this analysis.

## Discussion

We used logistic regression to test the relative impact of expression and evolution of genes from a diploid species, *S. tropicalis*, on the odds of functional persistence of duplicate genes generated by WGD in a tetraploid species, *X. laevis*. Our analysis employed EST databases and new molecular sequences from closely related outgroup species generated with next-generation sequencing of cDNA. We found that genes that are highly and broadly expressed, slowly evolving, and with more streamlined information packaging are preferentially retained as duplicates after WGD. Total level and evenness of expression have the largest impact of these parameters.

Higher and broader gene expression, both positively correlated with ohnolog retention, implies greater dosage sensitivity and pleiotropy, respectively [27,36,47-49]. The preferential retention of duplicate genes with these characteristics - stronger dosage constraints and more diverse interactions - supports the idea that expression dosage balance is important after WGD, and is consistent with other recent findings [13,24-27,45,50]. In addition, broader gene expression reduces the likelihood of duplicate gene loss through synfunctionalization [12]. The prediction that breadth is related to the level of pleiotropy could be further tested with protein interaction information, which is currently lacking for *Xenopus*.

Having higher and broader gene expression also allows more opportunities for partitioning paralogous expression patterns by subfunctionalization [2,51]. Here we did not test for evidence of expression subfunctionalization or neofunctionalization because comprehensive expression profiles in a closely related outgroup species are not available. But when one considers protein structure, higher information content in terms of longer genes and more exons could facilitate subfunctionalization, for instance because of a larger mutational target. In yeast, retained duplicates have longer proteins and more functional domains than singletons [52]. In contrast, we find that fewer exons and shorter intron length are more likely to be retained following WGD, after taking into consideration the effect of expression and molecular evolutionary rates. These findings suggest that subfunctionalized regions are generally not parceled by exons in *X. laevis*, or that protein subfunctionalization is subtle in this species.

It has been suggested that slowly evolving proteins may be preferentially subfunctionalized after WGD because they accumulate fewer substitutions over time than faster evolving genes, thus retaining their interchangeability for longer periods, and providing more opportunities for subfunctionalization to occur [23]. This is despite the observation that paralogs are often found to have higher evolutionary rates than singletons [53,54]. Our findings do not directly address this proposal, but we find that slower evolving genes (genes with lower dN and dS in *S. tropicalis*) were preferentially retained after WGD, which supports the expectations of Sémon and Wolfe [23].

Thus, in addition to supporting mechanisms based on dosage balance and pleiotropy, our results are also consistent with mechanisms for duplicate gene retention that involve regulatory subfunctionalization. The observation that gene expression has a major impact on the odds of functional persistence of ohnologs may be in part due to the greater mutational opportunities at the regulatory level compared with the protein-coding region. Once initial retention is achieved, for example through dosage balance or subfunctionalization, this may subsequently decrease constraints in different parts of the gene and thus allow further modifications to occur for long-term duplicate gene persistence [55,56].

Caveats exist in our interpretation of these data and results. We used genetic data from the most closely related diploid species available for comparison, which are not necessarily the same as the state of these genes in the diploid ancestor of the tetraploid species. However, we are only assuming that diploid characters are correlated with the ancestral state, and we argue that our interpretations are reasonable under the assumption that the relative rates of molecular evolution and expression between genes have generally remained similar since speciation (e.g. that broadly expressed genes in the extant diploid were also broadly expressed in the ancestral diploid). It is also possible that some duplicates were misidentified as singletons due to lacking data from one ohnolog or extensive divergence between ohnologs. We suspect that the proportion of misclassified singletons is small based on congruence between our database of triads and previous studies [23,43,57,58]. Because WGD in clawed frogs is probably the result of allopolyploidization (reviewed in [39]), these ohnologs presumably diverged to some degree in the diploid ancestral species before their contact in the tetraploid species. We suspect this does not have a large impact on our designation of dyads and triads, however our findings might thus be less representative of duplicate gene persistence in autopolyploids. It is also possible that segmental duplication occurred in some *S. tropicalis *loci, adding noise to our analysis. Again, we speculate that this would have a negligible impact on our overall conclusions and note that, using the UniGene sequence database, the *X. laevis *genes were always better BLAST hits for *S. tropicalis *sequences than any other *S. tropicalis *genes. We also expect the dataset of *S. tropicalis *orthologs to be quite comprehensive because a complete genome sequence is available for this species [59]. Additionally, our molecular data from dyads and triads could be biased towards slowly evolving genes with discernable homology to outgroup sequences from *P. carvalhoi *and *H. curtipes*. Whether or not this is the case is difficult to know without more complete data from the outgroup species. Another concern is that cloning biases and small EST sample size could impact expression estimates for some loci. While this is possible, it seems doubtful that this would substantially affect the magnitude of the correlation coefficients.

## Conclusions

We used logistic regression to evaluate the contributions of correlated genetic parameters in a diploid species on the odds of whether an ohnolog is retained in a closely related tetraploid species. Our results suggest that several parameters have a significant influence on the odds of duplicate persistence after taking other parameters into account, and that total expression and evenness of expression are most important. These findings are broadly consistent with retention mechanisms involving dosage balance and subfunctionalization. To further dissect apart the role of these retention mechanisms, one could examine patterns of expression and evolution of duplicate genes relative to an ortholog in an outgroup species. For example, studies of regulatory subfunctionalization in the tetraploid could determine whether the expression domain inferred for an ancestral gene are divided amongst the duplicate genes in the tetraploid [23,60,61]. A challenge in this type of study is the assessment of the ancestral expression state, because all orthologs have evolved since diverging from the most recent common ancestor. A possible alternative is to investigate protein interactions for reduced and partitioned pleiotropy among duplicates, but confirmation of subfunctionalization would also require inference of ancestral interactions.

## Authors' contributions

FJJC and BJE designed the study and drafted the manuscript. JD performed the statistical analyses and also participated in the design of the study and helped edit the manuscript. FJJC performed the data retrieval and molecular analyses. All authors read and approved the final manuscript.
